# Combining Thermal Effect and Mobility Control Mechanism to Reduce Water Cut in a Sandstone Reservoir in Kazakhstan

**DOI:** 10.3390/polym16121651

**Published:** 2024-06-11

**Authors:** Dilyara Sagandykova, Mariam Shakeel, Peyman Pourafshary

**Affiliations:** School of Mining and Geosciences, Nazarbayev University, Astana 010000, Kazakhstan; dilyara.sagandykova@nu.edu.kz (D.S.); mariam.shakeel@nu.edu.kz (M.S.)

**Keywords:** hybrid EOR, thermal EOR, polymer, water cut, Kazakhstan

## Abstract

The application of polymer flooding is currently under investigation to control water cut and recover residual oil from a giant sandstone reservoir in Kazakhstan, where the water cut in most producers exceeds 90%, leaving substantial untouched oil in the porous media. The primary objective of this research is to explore the feasibility of a novel approach that combines the mechanisms of mobility control by polymer injection and the thermal effects, such as oil viscosity reduction, by utilizing hot water to prepare the polymer solution. This innovative hybrid method’s impact on parameters like oil recovery, resistance factor, and mobility was measured and analyzed. The research involved an oil displacement study conducted by injecting a hot polymer at a temperature of 85 °C, which is higher than the reservoir temperature. Incremental recovery achieved through hot polymer injection was then compared to the recovery by conventional polymer flooding and the conventional surfactant–polymer-enhanced oil recovery techniques. The governing mechanisms behind recovery, including reductions in oil viscosity, alterations in polymer rheology, and effective mobility control, were systematically studied to comprehend the influence of this proposed approach on sweep efficiency. Given the substantial volume of residual oil within the studied reservoir, the primary objective is to improve the sweep efficiency as much as possible. Conventional polymer flooding demonstrated a moderate incremental oil recovery rate of approximately 48%. However, with the implementation of the new hybrid method, the recovery rate increased to more than 52%, reflecting a 4% improvement. Despite the polymer’s lower viscosity during hot polymer flooding, which was observed by the lower pressure drop in contrast to the conventional polymer flooding scenario, the recovery factor was higher. This discrepancy indicates that while polymer viscosity decreases, the activation of other oil displacement mechanisms contributes to higher oil production. Applying hybrid enhanced oil recovery mechanisms presents an opportunity to reduce project costs. For instance, achieving comparable results with lower chemical concentrations is of practical significance. The potential impact of this work on enhancing the profitability of chemically enhanced oil recovery within the sandstone reservoir under study is critical.

## 1. Introduction

The production of oil can be split into three main categories: primary, secondary, and tertiary enhanced oil recovery (EOR) techniques. Primary oil production relies on natural reservoir energy, allowing for the extraction of 10 to 30% of the oil originally in place (OIIP) using inherent forces to move oil towards production wells. When primary drive mechanisms fail to support optimal reservoir pressure for oil production, secondary recovery techniques such as water or gas flooding are employed, potentially increasing oil recovery by 10–30%. Water flooding, favored for its cost-effectiveness and widespread availability, is a frequently employed technique to augment oil recovery economically. However, the efficiency of water flooding is hindered by problems such as the higher mobility of the injection liquid than that of the oil, as a result of which crude oil is either not contacted by the injected water or is still stranded in shallow pores, leading to the common problem of high water production and high residual oil saturation [1,2].

To address the problem of low oil production by decreasing residual oil saturation and water cuts, several EOR techniques have been developed. One such technique is polymer flooding, which aims to increase sweep efficiency through an improved mobility ratio [3]. The injection of polymer solutions into reservoirs enhances the mobility control mechanism by substantially increasing the viscosity of the injection liquid, thereby ensuring uniform frontal displacement of the oil bank [4]. This front, developed by considerably viscous polymer fluids, redirects the flow of injected liquids from high-permeability areas to low-permeability regions that were previously bypassed or unswept. The polymer flooding EOR method ensures the mobilization of remaining oil after primary and secondary extraction by effectively reducing the mobility contrast between the injection liquid and hydrocarbons [5]. Polymer flooding has demonstrated success in various oil fields. For instance, the Daqing field in China, renowned for its large-scale polymer flood in a light oil reservoir, has shown an average additional oil recovery of approximately 12% of the original oil in place (OOIP). In 2019 alone, polymer flooding contributed to a total incremental oil recovery of 6.03 million tons. Another notable example is the Pelican Lake field in Canada, which boasts the world’s biggest polymer flood in heavy oil reservoirs and employs horizontal wells. Here, polymer injection has significantly augmented the recovery factor by more than 20% of OOIP for the flooded region, equal to 3.0 billion barrels. Additionally, this process achieves a low water-to-oil ratio, enhancing its effectiveness [6]. The polymer concentration is kept higher in the bulk phase owing to the expected decrease in in situ polymer concentration due to wettability alteration and polymer adsorption on the rock surface.

In addition to the mobility control mechanism of polymer flooding, there is another effective EOR method called surfactant flooding. Boosting oil production through surfactant flooding is achieved by diminishing interfacial tension (IFT) and promoting emulsification. Surfactants work by lowering the oil–water interfacial tension, facilitating improved contact and displacement of oil by injected liquids [7]. Additionally, surfactants are able to emulsify oil by dispersing it in water or the other way around, forming tiny oil drops in the aqueous phase, generally equivalent to or less than pore throats in size [8]. The effective displacement of these small drops through the rock enhances oil flow. While bigger droplets may lead to pore blockage and trapping, driving the surfactant formulation to bypassed areas enhances the volumetric sweep efficiency [5]. These mechanisms collectively lead to increased oil production and improved oil displacement within the formation.

The integration of surfactant flooding and polymer flooding (SP flooding) is a well-recognized hybrid chemical EOR method. By utilizing polymer flooding to enhance the viscosity of injection fluid for better mobility control and surfactant flooding to reduce the oil–water IFT, this combination method takes advantage of the benefits of both approaches [9,10]. There have been several successful instances of SP flooding in various oil fields. For example, at the Gudong field in China, which features an unconsolidated sandstone reservoir containing heavy oil, the oil production per day increased from 35 to 196 tons following the implementation of SP flooding. Similarly, at the Gudao field, also in China and characterized by high oil viscosity, the oil production rate progressively rose from 190 to around 870 tons per day, with an incremental oil recovery reaching 3.7% [11].

There is also a good alternative to the hybrid EOR technique for increasing oil production, particularly in the case of highly viscous or heavy oil, which involves integrating thermal and chemical flooding techniques. The viscosity of oil is temperature-dependent, leading to a decrease in oil viscosity as temperature increases [12]. The injection of hot water can modify the mobility ratio between oil and water by reducing oil viscosity and improving oil mobility, subsequently lowering the remaining oil saturation and resulting in higher recovery factors. The successful application of hot water flooding has been proven on both the lab scale and the field scale. From lab-scale research, displacement experiments conducted on one-foot Berea sandstone composite cores showed that the hot water flood produced a recovery of 49.62%, which was substantially greater than the 40.0% recovery achieved with the cold water flood [13]. Field-scale research demonstrated the successful application of hot water flooding in the Pelican Lake Field in northern Alberta, Canada. Oil production increased from approximately 6 m^3^/day to more than 25 m^3^/day during the hot water injection stage, and this level remained relatively steady for over two years [14]. Furthermore, a research study found that the IFT between oil and brine decreases with rising temperatures in heavy oil–brine reservoirs in the Niger Delta [15]. This can be explained with the following equation, which relates the IFT at the liquid interface to the droplet shape:

(1) $$\sigma =\frac{∆\rho gR_{0}^{2}}{\beta }\;\;$$

where σ is IFT, ∆ρ is the difference in density of the two immiscible liquids, g is the acceleration due to gravity, $R_{0}$ is the radius of curvature at the droplet tip, and β is the shape factor. Thus, interfacial tension is influenced by the density difference between the two liquids in the reservoir. An increasing temperature will decrease the density of both brine and oil, thereby reducing the density difference between the two phases, which will lead to a decrease in IFT [16].

Polymer flooding enhances sweep efficiency through increased water viscosity, but may cause injectivity issues. Combining thermal and chemical flooding methods can improve oil production by using hot water to enhance polymer injectivity and utilizing the viscous polymer solution for enhanced mobility control. However, due to the complexities and challenges associated with hot polymer flooding, including potential thermal degradation of the polymer and the need for precise temperature control, its application is currently limited on both the laboratory and field scales. It is crucial to consider the potential decrease in oil recovery due to polymer thermal degradation resulting from the integration of these two methods, which would impact the polymer flooding efficiency [12,17]. Rego et al. observed that, for hot polymer flooding, 100 °C was the optimal temperature for oil recovery. However, at temperatures exceeding 100 °C, there was a significant decrease in recovery efficiency due to polymer thermal degradation [17].

Field A in Kazakhstan is a prospective candidate for a study aiming to investigate and recommend the use of previously discussed techniques to extract the remaining hydrocarbons. The lithology of Field A is sandstone containing paraffinic oil, with permeability in the range of 100 to 500 md and porosity ranging between 24 and 27%. The crude oil in the reservoir possesses a density of about 0.80 g/cc, and the viscosity of the oil is around 5 cp at a formation temperature of 63 °C. The asphaltene content of the crude oil ranges between 12 and 15 wt%, and it contains around 20 to 28 wt% paraffin. Moreover, the formation water in the field exhibits a high salt content at 120,000 ppm, characterized by a high percentage of monovalent and divalent ions.

Oil production started in 1967 with the flooding of Field A using cold Caspian seawater, leading to challenges such as asphaltene precipitation [18]. Initially, untreated seawater caused paraffin solidification, blocking oil channels and clogging injectors, resulting in increased water production. Corrosive deposits from the interaction of seawater salts and formation oil during secondary oil recovery contaminated surfaces and subterranean structures [18,19]. Subsequently, a hot water flooding project at 85 to 90 °C proved to be a more successful strategy, addressing previous issues with the recovery method. Implemented to solve oil recovery challenges, this hot water injection yielded a controlled water cut and achieved an oil recovery of around 37% by 1975, reaching a plateau production level of about 16 million tons [20,21]. Still, there was a substantial jump in the water cut, which increased from 25% to 54%, coupled with a considerable 36% decrease in oil output during 1976–1983. Field development instability during the last decade resulted in a notable increase in water injection and the highest water cut in the course of field development [20]. Presently, Field A faces a significant challenge due to an extensive water cut, reaching 90% throughout the field, and yet maintains a significant residual oil saturation of over 50%. This study aims to identify the most effective EOR technique capable of improving oil recovery in the target field by efficiently controlling water production and displacing residual oil.

Polymer flooding is specifically an extensively used and successful treatment for high water production [22,23]. Combining polymer flooding with other approaches would be beneficial to obtain the maximum additional oil recovery, considering the previously indicated synergies in mobility control, reduction in IFT, and thermal effects. For the successful selection of EOR methods for Field A, a comprehensive study on oil displacement was essential. The study aimed to evaluate whether compounds chosen through diverse static processes and selection criteria could effectively enhance oil recovery. Several EOR methods, including polymer flooding, surfactant injection, hot water flooding, and their potential hybrids, were examined on the basis of their ability to mobilize oil and improve the incremental oil recovery from the formation. Emphasizing oil displacement outcomes, the prime focus of this investigation was to identify the most efficient and innovative EOR design suitable for Field A.

The idea behind this study was to test and investigate EOR schemes for the target field that would result in cost savings. The novelty of this study lies in the thorough and careful evaluation and design of chemicals, as well as a comparison of each design in terms of incremental oil recovery for the lowest possible chemical consumption. The availability of an on-site injection fluid-heating facility makes hot chemical flooding schemes an attractive option for the target field, and thus, this study is centered upon the design and evaluation of hot flooding schemes with which the chemical consumption and incurred cost would be lowered.

## 2. Methodology

### 2.1. Brines

The formation water (FW) in Field A had a density of 1.1 g/cc and a pH of 6.75. Chemical solutions for the tests were prepared using Caspian seawater (SW) with a salt content of approximately 13,000 ppm. The compositions of both brines are outlined in Table 1. Prior to the experiment, filtration of both brines was carried out to remove any debris and solid fragments, a precautionary measure to prevent the blockage of core sample pores and ensure the accuracy of the results.

### 2.2. Crude Oil

Crude oil from the target field was utilized for the surfactant selection stage and for the various oil recovery experiments. At a reservoir temperature of 63 °C, the oil exhibited a viscosity of 8 cp. Notably, the crude oil contained substantial proportions of asphaltenes at around 13 wt% and paraffin at 20 wt.%. Additionally, under reservoir conditions, the oil had a density of 787 kg/m^3^. To ensure purity, the crude oil underwent filtration before use to eliminate minerals, rock particles, and other impurities.

### 2.3. Rock Samples

Field A formation samples obtained from the target reservoir zone were employed for adsorption experiments of the surfactant and polymer, as well as for coreflood tests. The physical characteristics of the samples utilized in the oil recovery experiments are presented in Table 2.

A vacuum saturator from Vinci Technologies was used to saturate the cores manually with FW. The pore volume (PV) of the sample was then obtained using Equation (2).

(2) $$PV=\frac{W_{wet}-W_{dry}}{\rho_{FW}}\;\;\;\;\;\;\;\;\;\;\;\;\;\;\;\;$$

where *W_dry_* is the weight of the dry sample, *W_wet_* is the post-saturation weight of the saturated sample, and $\rho_{FW}$ denotes the density of FW.

### 2.4. Chemicals

The study for EOR in Field A comprised two main chemicals: a polymer and a surfactant. During the chemical screening phase, several polymer and surfactant samples supplied by the operator were passed through various screening criteria to select the optimum polymer and surfactant for the target field conditions. The following section provides a concise overview of each constituent and outlines the respective screening procedures.

### 2.5. Polymer

Four polymers derived from hydrolyzed polyacrylamide (HPAM), provided by the operator of Field A, underwent screening to select the optimal polymer based on criteria such as tolerance to bacterial attacks, thermal stability, oxygen presence, and static adsorption tendency. The complete procedure for polymer screening is referenced in another study by Yerniyazov et al. [1]. The polymer ASP3, with a molecular weight of 7.6 × 10^6^ Dalton, an intrinsic viscosity of 12.9 dL/g, and a degree of hydrolysis of 6.4%, was finalized as a result of the polymer-screening study. The preparation of the polymer solution followed the API 63-specified process. To create a 75% vortex in the seawater, a high-speed magnetic agitator at 600 rpm was employed, with the powdered polymer added gradually to the vortex shoulder within 30 s. Subsequently, the stirring was maintained at a reduced speed of 150 rpm to inhibit solid particle coagulation and polymer solution degradation. Gentle stirring was continued for 2–3 h before allowing the solution to sit all night. Parafilm was used to cover the solutions to avoid possible errors in results due to evaporation.

### 2.6. Surfactant

Numerous surfactants and co-surfactant samples provided by the operator of the target field were assessed to select the most effective surfactant on the basis of aqueous stability, microemulsion generation, and IFT reduction. The surfactant selection has already been completed, and the detailed results have been reported in a recent surfactant screening study by Dauyltayeva et al. [24]. Based on the results, Soloterra as a surfactant and Alfoterra as a co-surfactant were chosen for further investigation. Soloterra is an anionic surfactant provided by Sasol Germany GmbH as a liquid with 19 wt% activity. Alfoterra is also an anionic surfactant with a pH in the range of 8 to 10 and a density ranging between 1.12 and 1.16 g/cm^3^. For the preparation of surfactant samples, the desired weights of the surfactant and/or co-surfactant were gradually mixed into seawater at a fixed stirring rate of 150 rpm to avoid foaming in the sample. The solution was mixed for 2 to 3 h so that surfactant molecules would completely dissolve in the aqueous phase. The samples were wrapped with parafilm and preserved in a dry environment to prevent contamination.

### 2.7. Surfactant-Polymer Solution

To prepare a solution of surfactants and polymer, the necessary quantity of the surfactant and/or related co-surfactant was precisely weighed and then gradually introduced into SW while stirring constantly at 200 rpm to avoid foaming. The mixture underwent stirring for 2 to 3 h to make sure the surfactant molecules were fully dissolved in the aqueous phase. Next, at 600 rpm, the required amount of dry polymer was gradually added over 30 s. The stirring speed was then reduced to 150 rpm to avoid damaging the polymer solution. The solution was then left to infuse overnight. To minimize evaporation and reduce potential errors, the samples were sealed with parafilm.

### 2.8. Oil Displacement Tests

**Figure 1:**
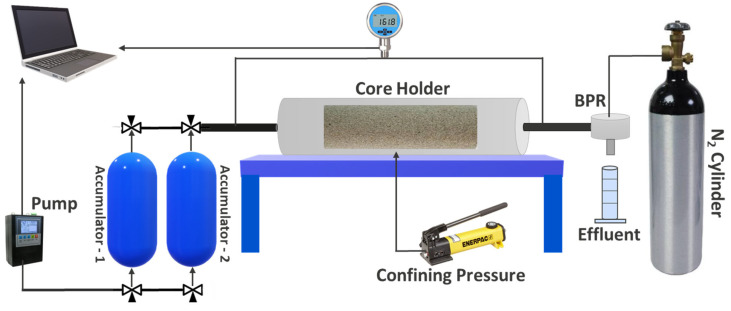
The graphic representation of the CFS-700 coreflood apparatus used for coreflood experiments [25].

After the selection of the chemicals from the methodical screening studies, a series of coreflood experiments was carried out to assess the oil recovery capability of individual and combined EOR schemes and to recommend the optimum EOR design for the target field. The tests were performed on Field A cores under reservoir conditions using the Vinci Technologies coreflood apparatus, CFS-700, which is schematically presented in Figure 1.

The steps listed below were followed during each coreflood test.

- FW was injected into the saturated core at varying flow rates, and the absolute permeability of the sample was calculated. The CFS system was prepared in accordance with the reservoir specifications at a temperature of 63 °C and a 1500 psi confining pressure to account for overburden pressure. The back pressure was maintained at 300 psi during the entire test except for polymer flooding. The back pressure at the time of polymer injection was set as equal to the atmospheric pressure to prevent polymer degradation in the outlet lines.
- Injection of crude oil was then initiated at 0.5 cc/min, which was then increased in increments of 0.5 cc/min whenever the water cut in the produced fluid fell below 0.1% at a particular flow rate. This was intended to minimize capillary end effects and to establish the initial water saturation (*S_wi_*) in the system. The criteria to switch the injection rate was a water cut of less than 0.1% in the produced fluid and a stable and consistent pressure drop across the core. Equation (3) was used to calculate *S_wi_* using the volume of the produced water in the effluents.
  (3) $$S_{wi}=\frac{PV-V_{w}}{PV}\times 100$$
  where *V_w_* is the amount of water produced in the effluents.
- Later, SW was injected into the core to obtain oil recovery through water flooding. Seawater flooding was conducted at 0.5 cc/min until the oil cut in the produced fluid was negligible and a stabilized pressure difference was established. The flow rate was then raised in increments of 0.5 cc/min to reduce capillary end effects and to ensure residual oil saturation by seawater injection (*S_orw_*). The produced oil volume during the SW injection stage was utilized to estimate the oil recovery with Equation (4).
  (4) $$Recovery\;factor=\frac{V_{oi}-V_{o}}{V_{oi}}\;*100$$
  where $V_{oi}$ is the volume of oil originally in place (OOIP) and $V_{o}$ is the oil volume recovered during water flooding.
- Subsequently, the designed chemical fluid at a specified strength was prepared in seawater, and injection was started at 0.5 cc/min. A similar criterion was set to change the injection rate to a higher value. The oil production obtained during this step was employed to determine the additional oil recovery through chemical injection. For thermal flooding methods, the CFS system was set at a temperature of 85 °C before injection.
- The resistance factor (RF), residual resistance factor (RRF), and capillary number (N_c_) were obtained using Equations (5)–(7), respectively.
  (5) $$Resistance\;Factor=\frac{{∆p}_{PF}}{{∆p}_{WF}}$$
  (6) $$Residual\;Resistance\;Factor=\frac{{∆p}_{WF}}{{∆p}_{postflush}}$$
  (7) $$N_{c}=\frac{K}{\sigma }{\left(\frac{∆p}{L}\right)}_{CF}$$
  where ${∆p}_{PF}$ is the pressure drop encountered in polymer injection, ${∆p}_{WF}$ is the pressure drop observed in water flooding, ${∆p}_{postflush}$ is the pressure drop established during seawater postflush, K denotes absolute permeability, ${\left(\frac{∆p}{L}\right)}_{CF}$ is the pressure gradient across the sample in a particular chemical injection phase, and σ is the oil–water IFT.
- Lastly, a seawater postflush was conducted to estimate RRF and to move the adsorbed chemicals out.

Similar steps were followed during all the coreflood experiments in order to find the most appropriate EOR technique for the target field.

## 3. Results

### 3.1. Chemical Screening

The first chemical ingredient to be investigated for the chemical flooding scheme in this research was a polymer. The polymer screening stage led to the selection of the HPAM polymer (ASP3) at a concentration of 2500 ppm. This choice was based on its ability to maintain the required viscosity of 5 cp during an extended thermal stability test. This high thermal stability ensures that the polymer can maintain its efficacy in improving oil displacement efficiency over extended operations. Furthermore, ASP3 exhibited favorable tolerance against non-ionic material present in seawater, particularly bacteria, and had a lower adsorption tendency on the Field A rock surface, as determined through static adsorption studies. The specifics of the polymer selection phase are reported elsewhere [1].

Following the polymer selection stage for Field A, the next chemical component to be assessed for the development of the chemical EOR recipe in this research was the surfactant. The selected formulation for the chemical flood design comprised the Soloterra surfactant (2S) and Alfoterra co-surfactant (2A) at a surfactant-to-co-surfactant ratio of 30/70 and a total strength of 1.5 wt%. This formulation demonstrated aqueous stability and generated Winsor Type III microemulsion, which consists of a surfactant-rich middle phase that coexists with excess oil and water phases and is usually advantageous for residual oil recovery due to its superior ability to reach ultra-low IFT values [26,27]. The further treatment of the generated emulsion involved the application of a thermal de-emulsification process to neutralize and eliminate the effects of emulsifying agents. This step was essential to separate the oil from the surfactant-rich and water-rich phases by disrupting the stability of the microemulsion, lowering the viscosity of the interfacial film, and consequently altering the IFT value [28,29]. Further optimization through IFT measurements revealed that the 1.5 wt% 2S/2A sample at a 30/70 mixing ratio reduced the IFT by two orders of magnitude. This formulation was thus selected for the subsequent stages of analysis in the chemical flood design process for Field A. The detailed results of the surfactant selection phase have already been reported in a previous study by our team [24].

### 3.2. Oil Displacement Tests

After selecting all chemicals for the Field A chemical EOR design, various hybrid methods involving blends of polymer flooding, surfactant injection, and hot water flooding were developed to assess their potential for enhanced oil recovery. Table 3 depicts the flooding sequence and the injection fluid recipes for the four oil displacement tests designed for the target field. The objective of the coreflood experiments was to quantify the combined effect of mobility control, reduction in IFT, and thermal effects using the screened chemicals. The aim was to select the optimum combination or hybrid EOR technique suitable for reducing the remaining oil saturation and establishing a uniform frontal advance to control the water cut.

### 3.3. Polymer Flooding

**Figure 2:**
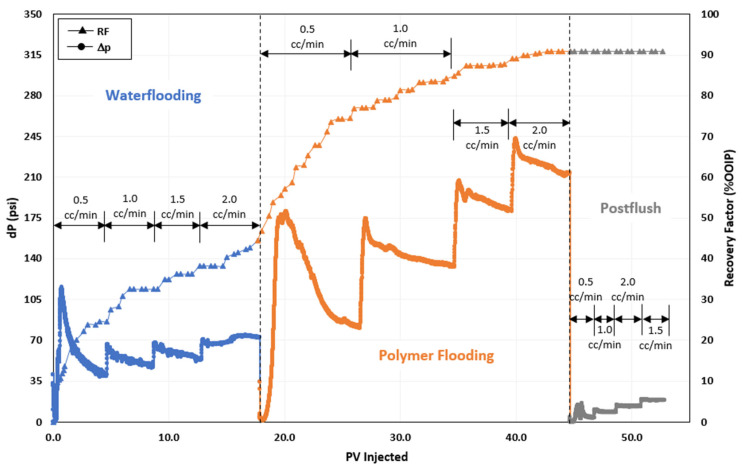
Oil recovery and pressure change profile of polymer injection test [25].

The first coreflood experiment in the sequence of oil displacement tests was flooding with the ASP3 polymer. Figure 2 presents the pressure change and oil recovery data for the entire experiment. The test began with the injection of seawater to reach a residual oil saturation after seawater injection (S_orw_). The recovery factor at the end of water flooding was approximately 42% of OOIP. The ASP3 polymer was then injected until there was negligible oil production in the effluent. PF produced around 48% incremental oil, raising the overall oil recovery to almost 91% OOIP. Ultimately, a seawater postflush was carried out to produce the unadsorbed polymer and to obtain the pressure change data for RRF estimation. The additional oil recovery achieved by PF can be accredited to displacing fluid mobility reduction and successful oil bank dislocation by the ASP3 polymer.

### 3.4. Hot Water Flooding

**Figure 3:**
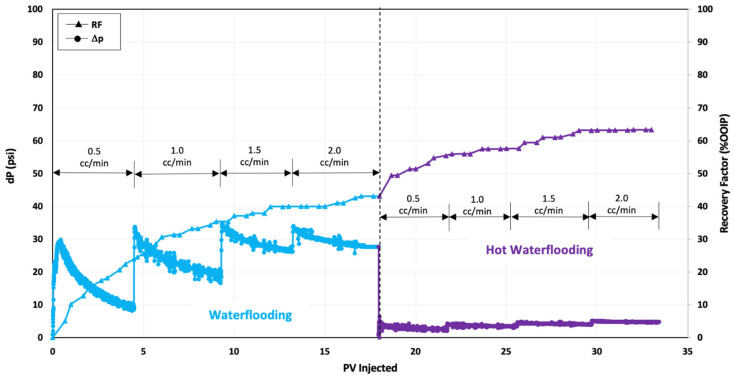
Oil recovery and pressure change profile of hot water injection test.

The subsequent oil displacement test aimed to assess the impact of thermal conditions on oil production by employing hot water flooding. Figure 3 depicts the data on pressure change and oil recovery from this test. Initially, a seawater (SW) injection was used to achieve residual oil saturation after water flooding (S_orw_). At the conclusion of the seawater injection, the oil recovery stood at approximately 43% of OOIP. Subsequently, hot water flooding began at 85 °C until no further oil was recovered in the effluent. This method yielded an additional 20% oil recovery, bringing the overall recovery factor to 63% OOIP. The enhanced oil recovery during hot water flooding can be attributed to the thermal effects, which reduced the viscosity of the oil. This reduction in oil viscosity was evident from the lower pressure drop values observed during hot water flooding compared to water flooding at the reservoir temperature of 63 °C.

### 3.5. Hot Polymer Flooding

**Figure 4:**
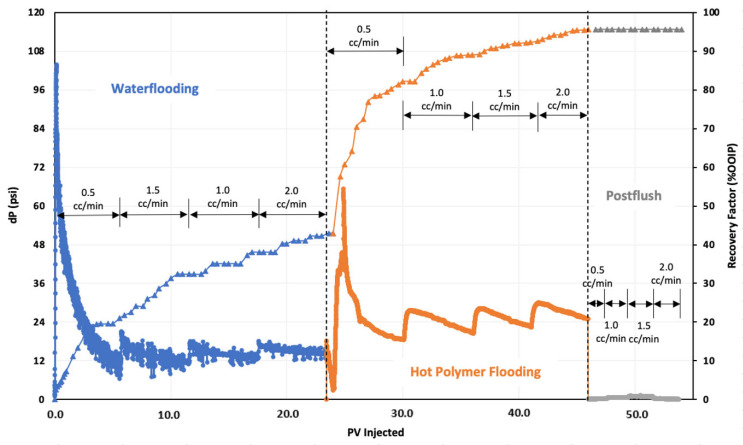
Oil recovery and pressure change profile of hot polymer injection test.

The second oil displacement experiment involved flooding with hot ASP3 polymer solution. This experiment was analogous to the PF test, except for the temperature at which the polymer was introduced during the chemical injection step. The temperature for this experiment was set to 85 °C. Figure 4 presents the oil recovery and pressure change data for the whole test. The oil recovery at the conclusion of the seawater injection step was around 42% of OOIP. The hot polymer injection was then commenced until the oil production in the effluent was negligible. Hot PF produced around 52% incremental oil, raising the overall recovery factor to 95% OOIP. Accordingly, the additional oil production by Hot PF was 4% higher compared to incremental recovery by conventional PF, and the pressure change at each flow rate was significantly lower during Hot PF. This decreased pressure drop behavior could be the result of reduced oil viscosity and better mobility control in the presence of thermal effects during polymer flooding.

The significantly lower pressure drop during hot polymer flooding shown in Figure 4 was due to several factors. Hot polymer flooding involves injecting the polymer solution at an elevated temperature, which reduces the viscosity of both the injected fluid and the reservoir oil. Lower-viscosity fluids flow more easily through the reservoir, resulting in lower pressure drops. Moreover, the higher temperature during hot polymer flooding can improve the sweep efficiency by reducing the mobility ratio between the injected fluid and the reservoir oil. This leads to better coverage of the reservoir and more effective displacement of oil, reducing the pressure drop required to push the oil toward production wells.

### 3.6. Combined Surfactant–Polymer (SP) Flooding

**Figure 5:**
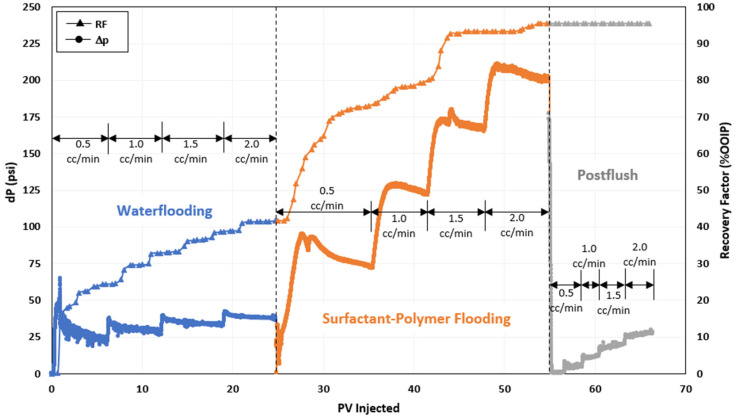
Oil recovery and pressure change profile of hybrid surfactant–polymer (SP) injection test [25].

**Figure 6:**
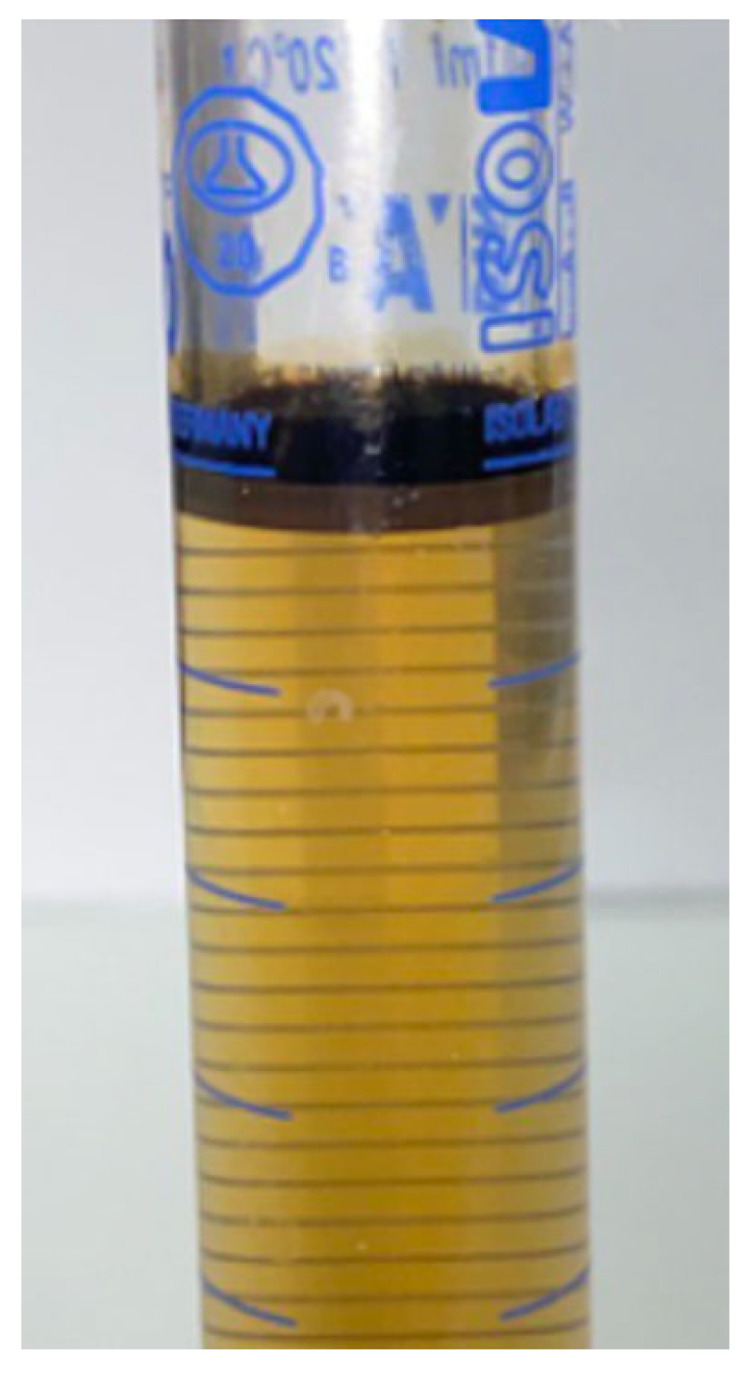
Winsor Type III microemulsion obtained during combined SP flooding [25].

After assessing the polymer efficiency, a hybrid of the polymer and the surfactant was evaluated to assess their combined action in producing residual oil. During the surfactant–polymer flooding test, a combined solution of the ASP3 polymer and the 2S/2A surfactant at an optimal concentration was utilized during the chemical flooding stage. Figure 5 shows the profile of the recovery factor and pressure drop for this test. In the combined surfactant–polymer (SP) flooding scenario, conventional water flooding initially resulted in the production of 42% of the original oil in place (OOIP). However, with the introduction of combined surfactant–polymer injection, there was a notable increase in oil recovery, leading to a total incremental oil production of 53% OOIP. The combined SP flooding schemes successfully generated Winsor Type III microemulsions in situ and effectively recovered the oil remaining after water flooding. Figure 6 illustrates the microemulsion produced during the chemical injection phase of this coreflood test, indicating the efficiency of combined SP flooding designs in generating microemulsions and reducing IFT. The fluctuations in pressure changes observed in Figure 5 may have been due to the clearing of pores, which were previously inaccessible by water during seawater injection, by injected fluids.

In the case of hybrid SP flooding, the surfactant requires some time to interact with crude oil to lower interfacial tension and consequently generate microemulsion, as was revealed by a lag in oil recovery due to the SP flooding experiment shown in Figure 5. A higher pressure difference in hybrid SP flooding can be caused by a higher residual oil saturation encountered by the injected chemical fluid. Owing to this high pressure difference, the injected SP formulation contacted a greater area of the pore space and released a greater saturation of residual oil.

### 3.7. Evaluation of Oil Recovery Performance of Various EOR Approaches

After the completion of flooding tests using various design options for Field A, an assessment of the effectiveness of various flooding methods in terms of oil recovery was carried out. The purpose of this assessment was to determine the most optimal hybrid flooding approach and to investigate how the combination of mobility control mechanisms and thermal effects affected enhanced oil recovery. Table 4 provides a summary of the oil recoveries observed during the different flooding stages of each oil displacement test. It was found that the secondary recovery was almost the same across all cores, as expected due to their similar formation and reservoir rock characteristics. Combinations of polymer flooding with thermal flooding and polymer flooding with surfactant flooding were tested to assess the synergy between mobility control, viscosity reduction, and IFT reduction mechanisms.

The results demonstrate the effectiveness of several EOR approaches, with incremental recovery factors ranging from 20% to 54% for various flooding schemes. Among all coreflood tests, hot polymer flooding and combined surfactant–polymer (SP) flooding were identified as the best EOR designs, achieving a recovery of 92% ROIC after water flooding.

The results indicate that hot polymer flooding and surfactant–polymer flooding achieved the highest incremental recovery factors at 52.7% and 53.8%, respectively. Polymer flooding alone yielded a slightly lower, but still significant, incremental recovery factor of 48.2%. The higher values of incremental recovery observed during hot polymer flooding are attributed to the combination of both mobility control mechanisms and thermal effects. The elevated temperature decreased the viscosity of the oil, enhancing its mobility, while also reducing the resistance to flow within the reservoir. This combined effect facilitated more efficient displacement of oil and contributed to the higher incremental recovery. In comparison, surfactant–polymer flooding achieved slightly higher incremental recovery due to the additional IFT reduction effect of the surfactant combined with the mobility control mechanism of polymer flooding. The surfactant lowered the IFT between the oil and water phases, allowing for better mobilization of trapped oil within the reservoir pores. This, combined with the mobility control provided by the polymer, enhanced the sweep efficiency and overall oil displacement, resulting in a slightly higher incremental recovery compared to hot polymer flooding.

**Figure 7:**
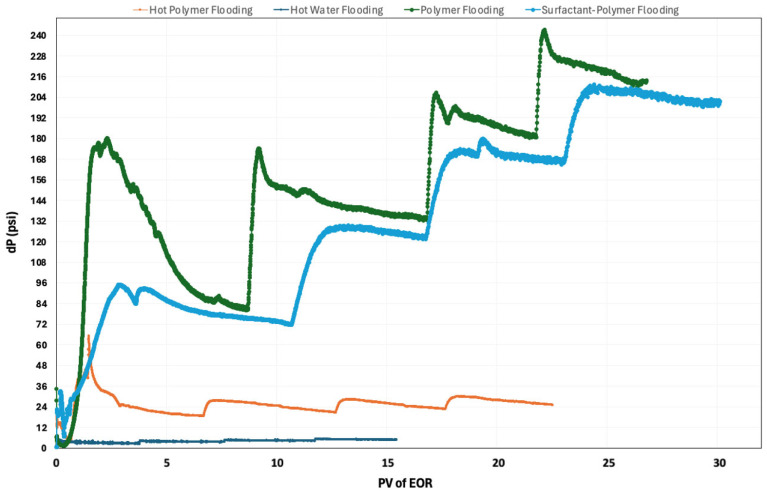
Pressure changes for different thermal/chemical flooding techniques.

In addition to the recovery factor, pressure changes can also be used to evaluate the incremental recovery across the different flooding techniques. Figure 7 above illustrates the pressure change results for four chemical flooding techniques. Polymer flooding typically experiences higher pressure changes due to the increased viscosity of the injected fluid, necessitating higher injection pressures to overcome reservoir resistance. Surfactant–polymer flooding, however, exhibits slightly lower pressure drops, reflecting the reduced resistance facilitated by surfactants. The combined effect of surfactants and polymers enhances sweep efficiency, requiring less pressure to overcome resistance. Hot polymer flooding, despite utilizing elevated temperatures, tends to result in a lower pressure drop compared to conventional polymer flooding. The increased temperature reduces the viscosity of both the injected fluid and the oil in the reservoir, facilitating improved fluid mobility and reducing the overall pressure drop required for displacement. Hot water flooding generally exhibits the lowest pressure drop among the four techniques. This is primarily due to the absence of added chemicals, resulting in a fluid with lower viscosity compared to polymer or surfactant–polymer solutions. Additionally, the thermal effects of hot water can further decrease the resistance within the reservoir, allowing for more efficient displacement with minimal pressure drop.

**Figure 8:**
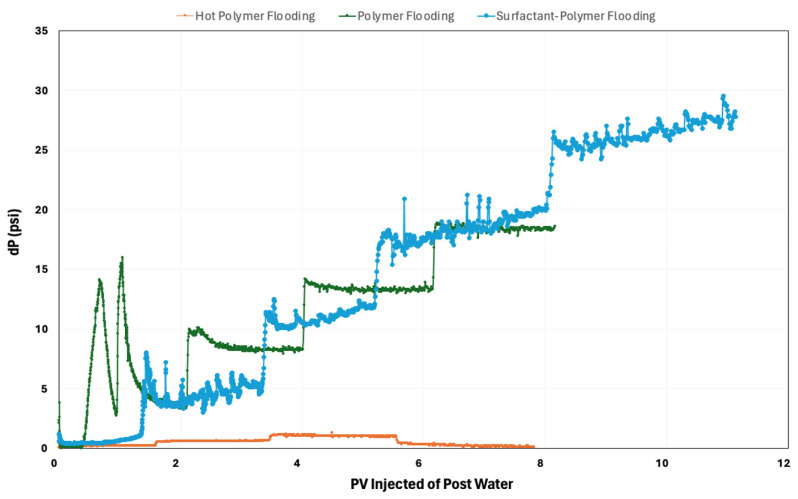
Pressure changes during postflush for different thermal/chemical flooding techniques.

Analyzing pressure drops during postflush experiments with seawater offers valuable insights into mobility control mechanisms. Figure 8 below represents the pressure change during postflush results for different chemical flooding techniques. Surfactant–polymer flooding typically exhibited the highest postflush pressure drop, followed by polymer flooding, and then hot polymer flooding. This trend suggests that surfactant–polymer flooding induced the most significant mobility control and flow diversions, resulting in increased resistance to flow postflush. This better mobility control ability can lead to improved sweep efficiency and enhanced oil displacement. In contrast, both polymer flooding and hot polymer flooding show lower postflush pressure drops, indicating relatively less pronounced mobility control. While polymer flooding alone still offers some mobility control, the addition of surfactants in surfactant–polymer flooding promotes IFT reduction, generating in situ microemulsions and further improving displacement efficiency by the polymer. Hot polymer flooding, despite its thermal effects, may induce less significant mobility control compared to surfactant–polymer flooding due to the absence of surfactants.

## 4. Conclusions

The comprehensive evaluation of various EOR techniques for Field A in Kazakhstan highlights the effectiveness of combining thermal and polymer flooding as a cost-effective alternative to conventional surfactant–polymer flooding. While SP flooding exhibits superior results in IFT reduction and mobility control, the evidence gathered suggests that the combined hot polymer flooding approach offers comparable incremental oil recovery rates while mitigating the financial burden associated with SP flooding.

The study examined the performance of polymer flooding, hot water flooding, hot polymer flooding, and combined surfactant–polymer flooding through oil displacement tests under reservoir conditions. The results indicate that hot polymer flooding and surfactant–polymer flooding achieved the highest incremental recovery factors, with values of 52.7% and 53.8%, respectively, surpassing the recovery achieved by polymer flooding alone. Hot polymer flooding capitalizes on the combined benefits of mobility control mechanisms and thermal effects, leading to improved oil displacement and higher recovery rates. Similarly, surfactant–polymer flooding demonstrated slightly higher incremental recovery due to the synergistic effects of polymer mobility control and surfactant IFT reduction. These recovery factors represent significant improvements over polymer flooding alone, which still yielded a substantial incremental recovery factor of 48.2%.

While SP flooding shows superior results in mobility control and IFT reduction, the study acknowledges that the combined thermal–polymer approach may induce less significant mobility control. However, considering the substantial cost savings associated with hot polymer flooding compared to SP flooding, the study concludes that hot polymer flooding presents a practical and cost-effective alternative for Field A in Kazakhstan.

## Figures and Tables

**Table 1:** Ionic composition of brines used in the study.

Ions	Ionic Strength (ppm)	
	Seawater	Formation Water
Na^+^	3514	18,900
Ca^2+^	401	3310
Mg^2+^	791	973
Cl^−^	6027	36,160
(SO_4_)^2−^	3140	76
HCO_3_^−^	255	390
K^+^	88	277
CO_3_^2−^	36	390
Total	14,248.7	60,083.0

**Table 2:** Physical characteristics of the rock samples used for oil recovery experiments.

Test	Sample ID	Length (cm)	Diameter (cm)	PV (cm^3^)	Porosity (%)	Permeability (md)
Polymer Flooding (PF)	1	5.55	3.79	15.2	24.3	323.1
Hot Polymer (Hot PF) Flooding	2	5.59	3.78	16.5	26.2	441.6
Combined Surfactant–Polymer (SP) Flooding	3	5.43	3.79	14.7	24.1	314.5
Hot Water (Hot WF) Flooding	4	5.55	3.76	14.8	24.0	352.3

**Table 3:** Design and injection fluid recipes for coreflood experiments.

Experiment ID/Name	Injection Sequence/Design	Remarks
Polymer Flooding (PF)	SW → Polymer → SW-Postflush	ASP3 polymer chosen on the basis of preliminary screening was utilized at an optimal concentration of 2500 ppm at a reservoir temperature of 63 °C.
Hot Water (Hot WF) Flooding	SW → Hot SW	Injection of SW at reservoir temperature of 63 °C, followed by injection of SW at a higher temperature of 85 °C.
Hot Polymer (Hot PF) Flooding	SW → Hot Polymer → SW-Postflush	ASP3 polymer chosen on the basis of preliminary screening was utilized at an optimal concentration of 2500 ppm and a temperature higher than the reservoir temperature of 85 °C.
Combined Surfactant–Polymer (SP) Flooding	SW → Surfactant–Polymer → SW-Postflush	A hybrid mixture of 1.5 wt% 2S/2A at a 30/70 mixing ratio and 2500 ppm ASP3 polymer was utilized.

**Table 4:** Summary of oil recovery performances of various chemical injection schemes.

Test Type	Flooding Stage	Recovery Factor	Incremental RF	
		(%OOIP)	(%OOIP)	(%ROIC)
Polymer Flooding	SW	42.8	-	-
	Polymer	90.9	48.2	84.1
Hot Polymer Flooding	SW	42.9	-	
	Hot Polymer	95.6	52.7	92.3
Hot Water Flooding	SW	43.1	-	
	Hot SW	63.4	20.3	35.6
Combined SP Flooding	SW	41.8	-	
	Surfactant–Polymer	95.5	53.8	92.3

## Data Availability

The data presented in this study are available on request from the corresponding author. The data are not publicly available due to restrictions imposed by the company involved in the project, which does not allow the authors to make the data public.
